# Transforming Waste Poly(Ethylene Terephthalate) into Nitrogen Doped Carbon Nanotubes and Its Utility in Oxygen Reduction Reaction and Bisphenol-A Removal from Contaminated Water

**DOI:** 10.3390/ma13184144

**Published:** 2020-09-17

**Authors:** Vadahanambi Sridhar, Hyun Park

**Affiliations:** 1 Global Core Research Centre for Ships and Offshore Plants (GCRC-SOP), Pusan National University, Busan 46241, Korea; sridhar@pusan.ac.kr; 2 Department of Naval Architecture and Ocean Engineering, Pusan National University, Busan 46241, Korea

**Keywords:** waste plastic, recycling, carbon nanotubes, microwave, electro-catalysts

## Abstract

Till date, waste plastics are either down-cycled to cheap products like fibers or burnt in incinerators to generate heat. In this manuscript, we report a simple and effective technique for microwave induced transformation of waste polyethylene terephthalate (wPET) to carbon nano-tubes (CNT). Iron nano-particles dispersed on graphene substrate acted as catalyst for CNT growth whereas urea served the dual role of de-polymerisation of wPET and also as nitrogen doping agent. Application of our newly synthesized 3-D meso-porous graphene-nitrogen doped carbon nanotube- iron electrode (Fe@NCNT-rGO) as electro-catalyst for oxygen reduction reaction (ORR) shows a positive half-wave potential (E_1/2_) of 0.75 V vs. RHE (reversible hydrogen electrode), nearly ideal four-electron pathway and excellent methanol tolerance when compared to commercial 20% Pt/C. The utility of Fe@NCNT-rGO for removal of bisphenol A from contaminated waters is also reported.

## 1. Introduction

Over 300 million tons of plastics are consumed every year with per capita consumption in excess of 100 kg in developed countries and around 20 kg in developing world. Of the many plastics used polyethylene terephthalate (PET) is the most common packaging material especially for beverages and water packaging and constitutes about 8% of solid urban waste [1]. Besides this, the consumption of PET is steadily increasing at a rate of 6 wt% annually and PET’s inherent non-biodegradability has created serious environmental and economic problems for urban waste managers. Though amongst all the common plastics used, due to its thermoplastic nature waste-PET (wPET) can be re-processed and re-used, but it is generally unsuitable for beverage packaging since the antimony catalyst and phthalate plasticizers commonly used in polymerization reaction and processing of PET will get reactivated [2]. Consequently, most wPET is “down cycled” to cheap fibers and used in applications such as floor mats or as packaging material. One alternative approach pursued vigorously at the start of this century is conversion of waste to energy (WtE) or energy from waste (EfW). PET due to its carbon rich, cheap and homogeneous nature has received increasing attention in recent years, as a precursor for synthesis of gas and liquid fuels for electric cogeneration. But in recent days, environmentalists are questioning the efficacy of thermal WtE technique due to environmental concerns about emission of green house gases like CO_2_ and release of dangerous dioxins into the atmosphere [3]. More ever, critics argue that incinerating waste destroys valuable resources and hampers recycling. Therefore, there is need for development of effective recycling technologies other than incineration. 

Carbonization is an attractive way of recycling carbonaceous wastes into useful nano-structured carbons. Carbonization can be broadly defined as the conversion of carbon rich substance into engineered carbons either by pyrolysis, destructive distillation or by hydrothermal methods. Hydrothermal carbonization is prevalent in nature and most of the solid fuels like coal, lignite, etc. are products of low-temperature carbonization of animal and plant residues with reaction times spanning over millions of years. Since, the earliest description of hydrothermal carbonization of cellulose by Bergius [4], many plant and animal based precursors have been carbonized to engineered carbons. Agro-based precursors such as sucrose [5], glucose [6], fructose [7], common sugar [8], ligno-cellulosic biomasses like bio-refinery residue [9], furfural manufacture waste [10], tamarind wood [11], willow bark [12], olive pomace [13], etc. Besides there are reports on carbonization of polymeric materials such as polypyrrole [14], polymer blends like poly(acrylonitrile)/poly(vinylidene Fluoride) [15] and co-polymers like poly(aniline-co-p-phenylenediamine) [16], etc. have been converted to carbons and used in applications such as adsorbents for environmental remediation [17], secondary batteries such as lithium- and sodium-ion batteries, etc. With millions of tons of waste plastic available every year and with 4% annual increase in plastic consumption, waste plastics and especially waste PET (wPET) can be a good source for synthesis of carbons by carbonization. Carbonization of waste plastics can be broadly divided into two categories: low temperature hydrothermal carbonization (LT-HTC) wherein the temperatures are typically below 300 °C and reaction times are typically in the range of 3 to 4 h which yield products such carbon microspheres [18]. The second category is high-temperature hydrothermal carbonization (HT-HTC) wherein the reaction times are well below 1 h and temperatures are in excess of 800 °C resulting in engineered carbons such as carbon nanotubes (CNT) [19,20], activated carbons [21], etc. Since the earliest reports of synthesis of CNT from waste polypropylene [22] many reports have been published studying the effects of “processing” and “operating” variables on the yield of CNT. The effect of process variables such as the type of plastics e.g., polypropylene or polyethylene or PET and its source, virgin or recycled have been studied in great detail by Arena et al. [23], Mishra et al. [24], and Acomb et al. [25]. These seminal works suggested that: waste plastics can be a good source of CNT and secondly, the catalyst should be preferably anchored on a support. Till date, organically modified nano-clays have been the most prevalent catalyst support, the primary reason being the layered structure of nano-clay impedes the fast escape of gases by “tortuous path” and helps in retention of catalyst particles. Graphene, the newest allotrope of carbon, first isolated by Geim and Novosalev [26], which when combined with metals either as 0-D quantum dots [27], 1-D nano-particles [28], 2-D nano-tubes [29] or 3-D clusters such as nano-flowers [29] have been reported in applications such as catalysis [30], energy storage devices [31], sensors [32], etc. Of these, graphene-CNT hybrids have proven to perform excellently in energy storage devices such as super-capacitors [33], electrodes in sodium-ion batteries [34], catalysts for ORR reactions [35], etc. Based on above mentioned factors, in this manuscript we report facile catalytic conversion of wPET to CNT by using: iron nano-particles dispersed on graphene as catalyst; urea as the de-polymerization agent and also as the source of nitrogen moieties and microwave irradiation as the external energy source. The utility synthesized 3-D mesoporous graphene-nitrogen doped carbon nanotube-iron electrode (Fe@NCNT-rGO) as electro-catalyst for oxygen reduction reaction (ORR) and for removal of bisphenol A from contaminated waters is reported.

## 2. Experimental

### 2.1. Materials

99.5% pure graphite (grade-QSG) and PAN (polyacrylonitrile) derived carbon fiber was purchased from Samjung C&G (Gyeongsan, Korea) and Alibaba (Hangzhou, China), respectively. Reagent grade chemicals sulphuric acid (CAS Number: 7664-93-9, ACS reagent grade, 98% purity), hydrochloric acid (CAS Number: 7647-01-0, ACS reagent grade, 37 wt% in H_2_O), sodium nitrate (CAS Number: 7631-99-4, ReagentPlus grade, 99%), hydrogen peroxide (CAS Number: 7722-84-1, Reagent grade, 50 wt% in H_2_O), potassium permanganate (CAS Number: 7722-64-7, Puriss grade, 99%), iron acetate (CAS Number: 3094-87-9, Puriss grade, 99.99%) and bisphenol A (CAS Number: 80-05-7, Reagant grade, 99%) were purchased from Sigma-Aldrich, Seoul, Korea, and were used as received whereas wPET was obtained from discarded PET soft drink bottles collected from local garbage boxes and cut manually into small pieces of approximately 10 × 10 mm^2^ and cleaned thoroughly with soap solution and DI water and dried.

### 2.2. Methods

Microwave oven manufactured by Daewoo Korea, Model number: KR-B202WL with output power of 700 W operating at 2450 MHz was used as the microwave source. Morphology of the carbon nano-structures were studied by Zeiss FEG-SEM Supra 25, Seoul, Korea (Field-emission scanning electron microscope operating at 10 kV and no metal coating was necessary since the synthesized carbon nano-materials showed excellent conducitivity). High resolution transmission electron microscopic images, high-angle annular dark-field (HAADF) images and nano-scaled elemental maps were recorded on TALOS F200X, Thermo Fisher Scientific Korea Ltd., Seoul, Korea operating at 200 kV. An Belsorp Mini II Surface Area analyzer, (Microtrac MRB, York, PA, USA) operating at −196 °C was used to measure the surface area and porosity. LabRAM high resolution confocal Raman spectrometer (Horiba France SAS, Longjumeau, France) was used to record Raman spectra and the electronic states of chemical moieties was studied by a Sigma Probe Thermo VG ray photoelectron spectrometer (Thermo Fisher Scientific Korea Ltd., Seoul, Korea). Deconvolution and curve fitting of XPS data was carried out using XPSPEAK4.1 (https://xpspeak.software.informer.com/4.1/).

Electrochemical tests were performed on an electrochemical station, CHI 660 manufactured by CH Instruments, Inc. Austin, TX, USA with a conventional three-electrode set up using a saturated calomel electrode (SCE) and Pt wire as the reference and counter electrodes, respectively and 0.1 M KOH aqueous solution as the electrolyte. Catalyst for all the electrochemical tests was prepared by dispersing 5 mg of Fe@NCNT-rGO dispersed ultrasonically for 30 min in 500 µL solution consisting of 480 µL of isopropanol (J&K Scientific Ltd, Seoul.) in which 20 µL of 5 wt% Nafion solution (DuPont) was dissolved. 12 µL of this catalyst suspension was pipetted onto a 5 mm diameter glassy carbon RDE (rotating disk electrode) or 4.93 × 5.38 mm inner and outer diameter RRDE (rotating ring-disk electrode) and the solvent was removed by drying naturally in air. To achieve a mass loading of 0.6 mg/cm^2^ the above steps were repeated twice. Prior to and also throughout the course of electrochemical tests, the electrolyte was saturated with O_2_. The potential values were converted to the reversible hydrogen electrode (RHE) scale, according to the equation: E_RHE_ = E_SCE_ + 0.0591 pH + 0.242 and reported in this study. In case of stability tests, experiments were carried out at rotation speed of 1600 rpm, operating voltage of −0.7 V vs. SCE for 10 min in 0.1 M KOH O_2_− saturated electrolyte. Commercial Pt/C catalyst with 20 wt% Pt was used for comparison tested under the same conditions, with a loading mass of 0.25 mg·cm^−2^. LSV (Linear sweep voltammetry) tests at 10 mV/s scan rate and rotating speeds ranging from 400 to 2400 rpm were carried out using RDE/RRDE.

### 2.3. Bisphenol-A Adsorption Studies

Adsorption isotherms of bisphenol A on Fe@NCNT-CF were obtained by adding 0.1 g of Fe@NCNT-CF to beakers containing 75 mL of bisphenol A solution at different concentrations (10−320 mg/L). The pH of the solutions was adjusted by adding dilute HCl or NaOH.

### 2.4. Converting Waste PET into Nitrogen Doped Carbon Nanotubes

The synthesis of 3D carbon nano tubes anchored on graphene was carried out in a “one-pot” microwave technique. Briefly, graphene oxide prepared by a modified Hummer’s method [31], iron acetate, urea and finely chopped wPET were mixed in weight ratio of 1:0.25:4:20 and subjected to microwave irradiation at 700 W for 300 s to obtain a fluffy powder. The obtained product was washed with ethanol and de-ionized (DI) water to remove traces of any un-reacted urea or wPET and further dried in an oven to obtain Fe@NCNT-rGO.

## 3. Results and Discussion

**Figure 1:**
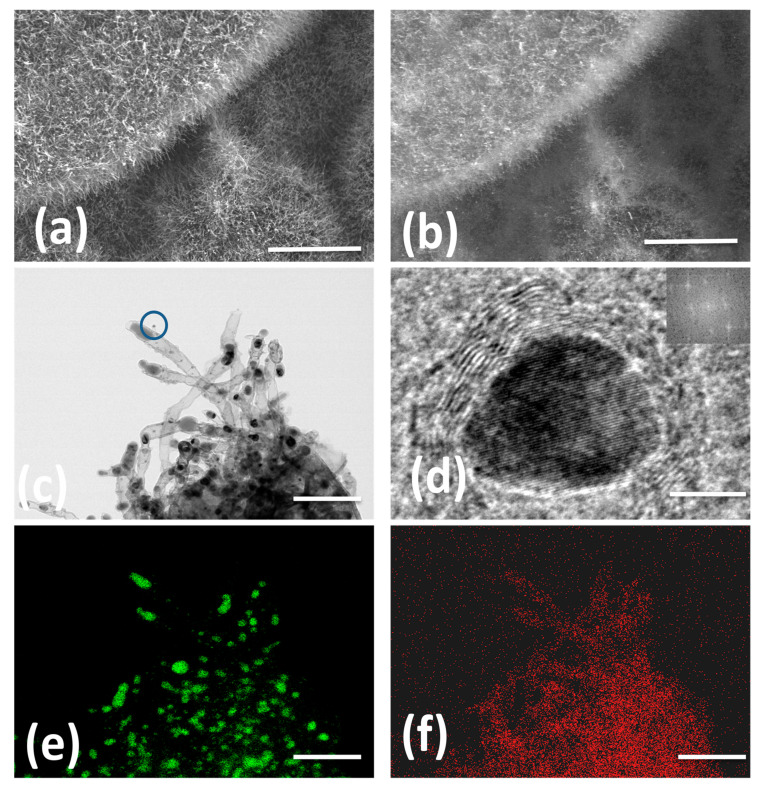
In-lens and secondary ion emission SEM micrographs of wPET derived short carbon nanotubes well distributed along the graphene substrate (**a**,**b**) HRTEM (**c**) at low and high magnification; Figures **e**,**f** are iron and nitrogen EDX maps. Scale bars are 3 µm, 3 µm, 300 nm, 10 nm, 300 nm and 300 nm in (**a**–**f**) respectively.

The morphology and microstructure of Fe@NCNT-rGO studied by in-lens and secondary ion emission SEM images exhibited in Figure 1a,b shows dense carbon nanotube with typical lengths in the range of 500 to 900 nanometers and are well distributed on the graphene substrate. Though in-lens mode of SEM imaging is widely prevalent, secondary-ion images are quiet useful in imaging “hidden” features. In our case, due to higher conductivity of graphene, they appear brighter when compared to CNT and iron moieties. Representative HRTEM micrograph exhibited in Figure 1c confirms the presence of 50–60 nm thick multi-walled CNTs with large sized iron nano-particles at the tip of the tubes and smaller iron nano-particles trapped either inside of the tubes or anchored on the walls of tube. This can be attributed to the catalytic activity of iron nano-particles. Tip growth and base growth are the two most prevalent models of CNT growth mechanisms. If the catalyst is anchored or fixed on a substrate, a base growth model is prevalent wherein short CNTs with catalyst particles at the base of the carbon nanotubes are generally observed. However, in cases where the catalyst is “free” and not pinned onto a substrate, a tip growth model is envisaged resulting in long, entangled and curved CNTs. Generally, chemically derived graphene (CDG) synthesized from graphite precursors are intrinsically endowed with chemical and physical defects attributed to oxidation and mechanical stresses generated from shear forces during ultra-sonication, respectively. These defects are advantageous and act as anchoring points for the catalyst iron nano-particles; thereby CNT growth predominantly follows a base growth mechanism in our case. The yield of CNTs calculated by weight gain method, is found to be 22.87 ± 0.5 wt%.

In order to test the utility of wPET to grow CNT with other catalysts, commercially palladium on carbon (with 30% of Pd) was used as the catalyst, CNT clusters with morphology similar to “St. Augustine grass” and catalyst nano-particles well distributed along the length and breadth of tubes were observed (Figures S1 and S2 in supplementary file). Figure 1d shows high-resolution HRTEM image of a single iron oxide nano-particle of 12 nm size with a characteristic interlayer distance of 0.26 nm, which is ascribed to the (311) lattice fringe of Fe_3_O_4_. Representative EDX elemental mappings of N and Fe exhibited in Figure 1e,f, respectively, shows that the addition of urea imparts nitrogen doping of synthesized CNT and is uniformly distributed all along the walls of nanotubes. Corresponding HAADF image is exhibited as Figure S3 in supplementary file.

**Figure 2:**
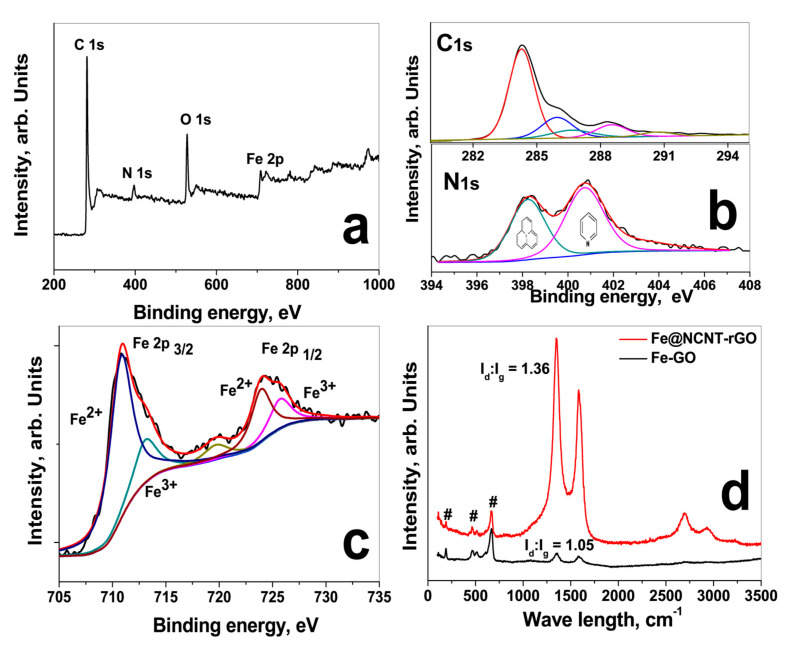
XPS survey scan (**a**); Deconvoluted C 1s spectra of GO (upper part) and Fe@NCNT-rGO (lower part) in (**b**); deconvoluted Fe 2p spectra (**c**) and Raman spectra (**d**).

In order to further study the chemical structure of C, N and Fe moieties, X-ray photoelectron spectroscopy (XPS) was carried out and Figure 2a shows the survey scans in the range of 0 to 1000 eV. In Fe@NCNT-rGO, in addition to C 1s and O 1s peaks, there is a Fe 2p peak in the range of 705 to 735 eV with additional minor peaks at 90–110 and 45–65 eV, attributed to Fe 3s and Fe 3p, respectively. The deconvoluted C 1s XPS spectra shows a predominant peak at 284.5 eV attributed to the C-C and C=C and three minor peaks at (i) 285.98 eV attributed to carbon singly bound to nitrogen; (ii) the peak at 286.9 eV attributed to N-sp^2^-C configurations; (iii) minor peak at 288.3 eV due to sp^3^ trigonal CN bonds, and the peak at 290.7 eV attributed to the π-π shake up. The deconvoluted N 1s peak of G-NCNT-Fe is shown in lower part of Figure 2b indicates the presence of two major nitrogen species differentiated by their binding energies (BE); pyridinic (BE ≈ 398.2 eV) and pyrrolic (BE ≈ 400.2 eV). Quantitative analysis based on peak areas show that the nitrogen moieties are 39% pyridinic, 61% pyrrolic. In order to study the chemical composition and structure of iron nano-particles, high resolution XPS spectra of Fe 2p in the range of 878 to 850 eV were recorded and the deconvoluted spectra is plotted in Figure 2c which shows two distinct peaks at centered at 710.9 and 724.4 eV corresponding to Fe 2p_3/2_ and Fe 2p_1/2_ and separated by a distance of 13.5 eV due to split spin-orbit components. Besides these, two satellite peaks at 713.2 and 725.8 eV are also observed indicating that the iron moieties exist in Fe^3+^ state in the form of iron oxide, Fe_3_O_4_.

The Raman spectra of iron-decorated graphene and Fe@NCNT-rGO Figure 2a were tested at a laser excitation frequency of 514 nm. Three peaks at 189, 468, and 670 cm^−1^ corresponding to iron moieties are observed in iron-decorated graphene and Fe@NCNT-rGO in addition to the well-known Raman active G band peak of graphene at 1583.2 cm^−1^ attributed to the in-plane vibrational mode and 1350.6 cm^−1^ peak assigned to the disorder induced by the presence of CNTs on the graphene substrate. Besides in Fe@NCNT-rGO, the 2D peak occurring in the vicinity of 2600 cm^−1^ is sharper and it is related to sp_2_-carbon bonds combined with a minor highly ordered pyrolytic graphite (HOPG) hump occurring at 2400 cm^−1^. From XPS and Raman studies it can be concluded that iron is predominantly in Fe^3+^ state in the form of iron oxide, Fe_3_O_4_ with some minor quantities of Fe-Nx moieties. Additionally, a substantial increase in the ratio of intensities of D band to G band (I_d_/I_g_ ratio) from 1.05 in G-Fe to 1.36 in Fe@NCNT-rGO can also be observed which indicates partial healing of in-plane structural defects induced in graphene by oxidation and intense sonication during synthesis of graphene oxide.

**Figure 3:**
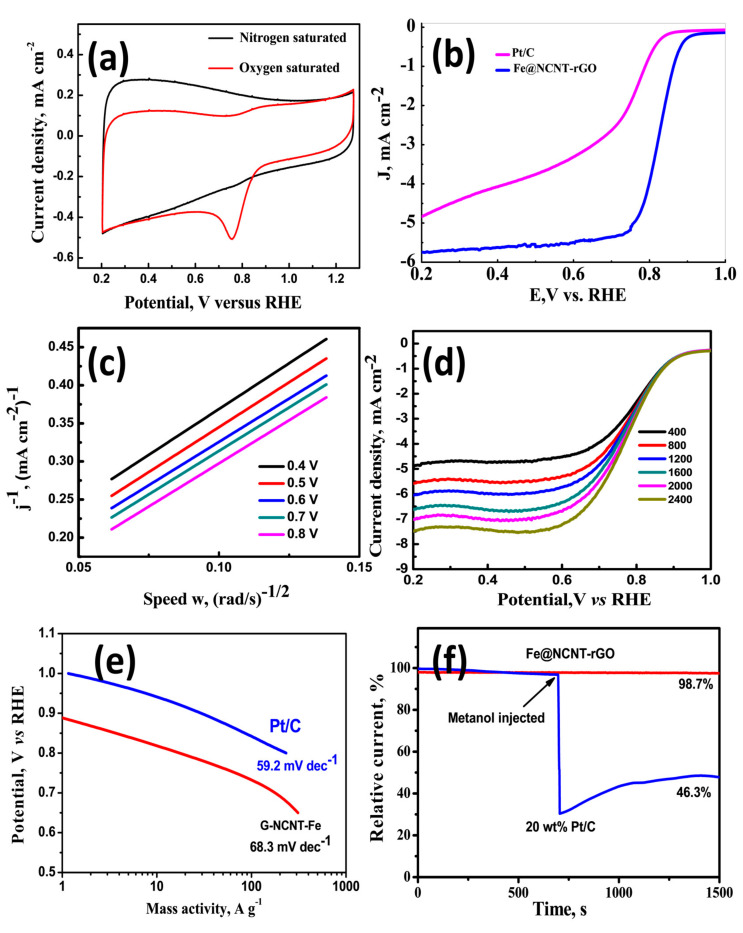
Current-Voltage curves of Fe@NCNT-rGO electrode in N_2_ (black) and O_2_ (red) saturated 0.1 M KOH electrolyte at a sweep rate of 50 mV s^−1^ (**a**); Comparative Linear sweep voltammograms of commercial 20 wt% Pt/C and Fe@NCNT-rGO in 0.1 M KOH at a rotation rate of 1200 rpm (**b**); Koutecky–Levich (K–L) plots of Fe@NCNT-rGO at potentials of 0.4 to 0.8 V (**c**); Comparative Linear sweep voltammograms of Fe@NCNT-rGO various rotation rates (**d**); Comparative Tafel plots of Fe@NCNT-rGO electrode and commercial 20 wt% Pt/C (**e**); and Comparative methanol tolerance test of Fe@NCNT-rGO and commercial 20 wt% Pt/C (**f**); in 0.1 M KOH.

The electrochemical behavior as studied by CV diagrams (Figure 3a) of our newly developed wPET derived Fe@NCNT-rGO was tested in nitrogen and oxygen saturated 0.1 M KOH electrolytes. Irrespective of the saturation, the CV curve has similar shape of our Fe@NCNT-rGO is similar to that of other reported Fe-N containing carbon nano-allotrope electrocatalysts [36,37,38] and the CV curve in oxygen saturated system shows a visually discernible peak at ~0.755 V whereas in nitrogen saturated electrolyte this peak is not observed which indicates the our newly developed wPET derived Fe@NCNT-rGO are excellently electro-catalysts for ORR type reactions. The ORR activity and kinetics was studied by linear sweep voltammetry (LSV) tests using a rotating disk electrode (RDE) in an O_2_ saturated 0.1 M KOH electrolyte and compared with that of commercially available 20 wt% Pt/C electrodes and plotted in Figure 3b which indicates that Fe@NCNT-rGO exhibits a distinct electrocatalytic ORR activity with a high onset potential of 0.896 V vs. RHE, which is close to the onset potential potential of commercial Pt/C catalyst (0.92 V). Furthermore, our Fe@NCNT-rGO exhibits considerably lower current density of 4.8 mA cm^−2^ when compared to 5.7 mA cm^−2^ observed in commercial Pt/C electrodes. Besides, the wide voltage range (from 0.22 to 0.76 V) indicates that the reaction kinetics is predominantly controlled by diffusion [39]. The kinetic parameters of the ORR reaction were studied by Koutecky-Levich (K-L) plots and the RDE polarization curves at rotation speeds from 400 to 2400 rpm are plotted in Figure 3c. At all measured voltages from 0.2 to 0.7 V, the K-L plots of wPET derived Fe@NCNT-rGO electrodes shows excellent parallel linearity indicating first-order reaction kinetics of iron and nitrogen containing ORR carbo-catalysts [39,40]. The number of electron transfer (n) per oxygen molecule calculated by K-L equation is 3.8 which is close to the theoretical value of 4.0 for commercial Pt/C, and reiterates that a nearly four electron pathway occurs in our newly developed Fe@NCNT-rGO electrodes. A comparative Tafel plot which is typically associated with the oxygen adsorption behavior of our Fe@NCNT-rGO and commercial 20 wt% Pt/C plotted in Figure 3e shows that the slope of (68.3 mV dec^−1^ vs. 59.2 mV dec^−1^), suggesting an Eley-Rideal mechanism [41], wherein under an applied voltage, water molecule in basic solution breaks its OH bond over surface active sites on the catalyst surface with concurrent formation of O-OH. The chemical composition of our wPET derived Fe@NCNT-rGO as studied by XPS survey spectra shows C: 87.47, N: 4.56, O: 1.17 and Fe: 6.8 wt% which is present either as Fe-N_x_ nano-particles embedded inside and along the walls of CNTs and the also nitrogen moieties distributed along the walls of bamboo shaped CNTs which thereby enables efficient catalytic activity and enhancing the mass transfer of ORR-related species.

For most practical purposes, the phenomenon of methanol crossover tolerance in direct methanol fuel cells (DMFCs) is a major hurdle which not only reduces the ORR activity but alos reduces the efficiency of the fuel cell. Therefore a typical ORR catalyst is expected to show considerable resistance to methanol posining. The methanol resistance of our Fe@NCNT-rGO and commercial 20 wt% Pt/C was tested by chrono-amperometric tests and plotted in Figure 3f which shows there is only marginal decrease to 98.7% in relative current (I/I_0_), when compared to more than 50% decrease in 20 wt% Pt/C. This can be attributed to the embedding of the catalytically active Fe-Nx moieties inside the carbon nanotubes; along the walls of CNT in which the nano-sized Fe-Nx cores are enclosed in a protective carbon core thereby preventing oxidation and consequent fouling of catalyst due to the crossover of methanol. The above stated properties like excellent onset potential, better Tafel slope and almost no adverse effect of methanol on relative current makes our Fe@NCNT-rGO electrodes an attractive alternate non-platinum group metal catalyst for ORR reactions.

**Figure 4:**
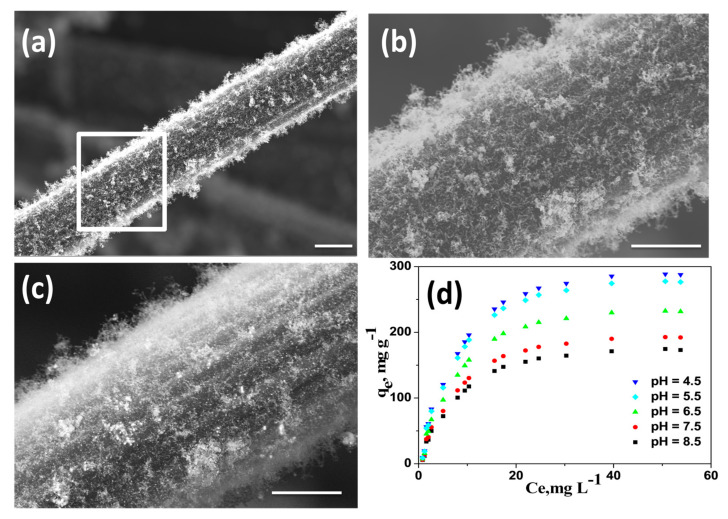
Representative SEM micrographs of Fe@NCNT-CF hybrid synthesized by from wPET at low (**a**) and high (**b**) magnifications; (**c**) is the secondary electron image showing presence of iron nano-particles; Scale bars are 50 µm in (**a**) and 1 µm (**b**) and (**c**); Adsorption isotherms of bisphenol A on Fe@CNT-CF nanostructures at various pH (**d**).

We also investigated the utility of our newly developed wPET/Urea precursors to grow CNT on other carbonaceous substrates like carbon fiber (CF). Amongst the various available microwave susceptible materials, carbon fiber was chosen due to the fact that it can endure short time microwave radiation without undergoing extensive structural distortions. Commercially available CFs purchased from local market were first finely chopped into 0.5 cm length, washed successively with 10 wt% aqueous HCl and DI water to remove the commercial “sizing” materials and mixed with wPET/Urea. Subsequently this mixture was subjected to microwave irradiation for 60 s to obtain nitrogen doped CNT grown on carbon fibers (Fe@NCNT-CF) and a representative SEM micrographs recorded without any metal conductive coating at low and high resolutions are exhibited in Figure 4a,b, respectively. High density growth of CNTs along the length of carbon fibers can be observed with the length of CNTs in the range of micrometers. The secondary electron image shown in Figure 4c shows the iron metal nano-particles are well dispersed and are predominantly distributed amongst the CNT. This result shows the versatility of our newly discovered wPET/Urea precursors as excellent source to grow CNTs on a wide range of microwave susceptible substrates, the only caveat being the substrate should be capable of withstanding physicochemical damage due to short-term microwave radiation.

The applicability of wPET/Urea derived Fe@NCNT-CF for the removal of bisphenol A from contaminated water is investigated. Bisphenol A [2,2-bis(4-hydroxyphenyl)propane or BPA] is an important high production volume chemical, with more than 12 million tons produced annually and extensively used in the production of polycarbonates and epoxy resins. BPA’s exposure to humans is widespread especially by usage of household plastic articles like water bottles, food storage containers (the so-called “Tupperware”), feeding bottles for babies, plastic toys, etc. [42]. BPA contamination has been detected in industrial wastewater, groundwater, surface water, and even drinking water. But BPA is one of the most common Endocrine-disrupting chemicals (EDC). An EDC is a chemical that imitates biological activity of natural hormones and occupies the hormone receptors, and because its weak estrogen-like effect interfere with the metabolic processes of natural hormones especially in females thereby posing a considerable risk to reproductivity in humans. Besides due to the biochemical activity of liver, BPA is converted to 4-methyl-2,4-bis(4-hydroxyphenyl) pent-1-ene (MBP) which has 1000-fold stronger binding affinity to endocrine receptors compared to BPA [43]. Though many techniques like adsorption, ion-exchange, membrane, oxidation, etc. are available for removal of BPA, adsorption by sorbents is the most effective and economical. Of the many available adsorbents, the most popular are carbon based adsorbents and wide range of carbon materials such as activated carbon [44], CNT [45], reduced graphene oxide [46], etc. have been investigated, but the utility of CNT-Carbon fiber hybrids has never been reported. This manuscript is the first ever report on the utility of iron oxide-CNT grown on carbon fibers as adsorbent of bisphenol A from contaminated waters. The adsorption isotherms of bisphenol A at acidic, neutral and basic pH values by our novel Fe@NCNT-CF adsorbent is plotted in Figure 4d which shows that at all investigated concentration of BPA and pH values, our Fe@NCNT-CF nanostructures showed wide range of adsorption capacities ranging from 150 to 300 mg/L, with maximum adsorption observed in acidic pH which is more than twice than the reported values in graphene based BPA adsorbents [47]. This high adsorption value can be attributed to two major types of absorption sites namely: the interstitial meso-pores between the vertically anchored CNTs and the defects on the walls of CNT due to nitrogen doping which facilitates the optimal capture and retention of BPA moieties. This result indicates that our newly developed Fe@NCNT-CF synthesized from wPET can be an excellent adsorbent for benzene containing organic pollutants.

## 4. Conclusions

In summary, we report the microwave synthesis of iron and nitrogen co-doped carbon nanotubes (Fe@NCNT-rGO) from wPET bottles using iron nano-particles anchored on graphene as catalyst, urea as the combined source of nitrogen moieties and de-polymerization agent whereas microwave radiation was external energy source. Morphological structural analysis by SEM and HRTEM reveals that our synthesized Fe@NCNT-rGO hierarchical carbon-nanostructures are composed of millimeter long, bamboo shaped and defect rich carbon nanotubes anchored on reduced graphene oxide substrate with nano-sized iron particles embedded on walls and the interstitial voids inside the carbon nanotubes. Elemental analysis by EDS maps show excellent and uniform distribution of nitrogen and iron moieties in carbon nanotubes which on further analysis by deconvolution of high resolution XPS data shows that the nitrogen moieties are predominantly in Fe-N-C, pyridine and pyrrole structures which boosts the electro-catalytic activity, durability and methanol tolerance of wPET derived Fe@NCNT-rGO when applied as catalyst in ORR reactions. The excellent ability of Fe@NCNT-CF for removal of bisphenol-A from contaminated waters at acidic, basic and neutral pH values is also demonstrated.

## Supplementary Materials

The following are available online at https://www.mdpi.com/1996-1944/13/18/4144/s1, Figures S1 and S2: SEM micrographs of CNT clusters grown using commercial palladium on carbon (30% Pd) showing “St Augustine grass” type of morphology. Figure S3: HAADF dark field image corresponding of G-NCNT-Fe corresponding to Figure 1c.
