# Supplementary material for: Transforming Waste Poly(Ethylene Terephthalate) into Nitrogen Doped Carbon Nanotubes and Its Utility in Oxygen Reduction Reaction and Bisphenol-A Removal from Contaminated Water
Source: Materials (Basel). 2020 Sep 17;13(18):4144. doi: 10.3390/ma13184144 (PMC7560256; doi:10.3390/ma13184144)
Supplement: Supplementary file 1 [file materials-13-04144-s001.pdf]

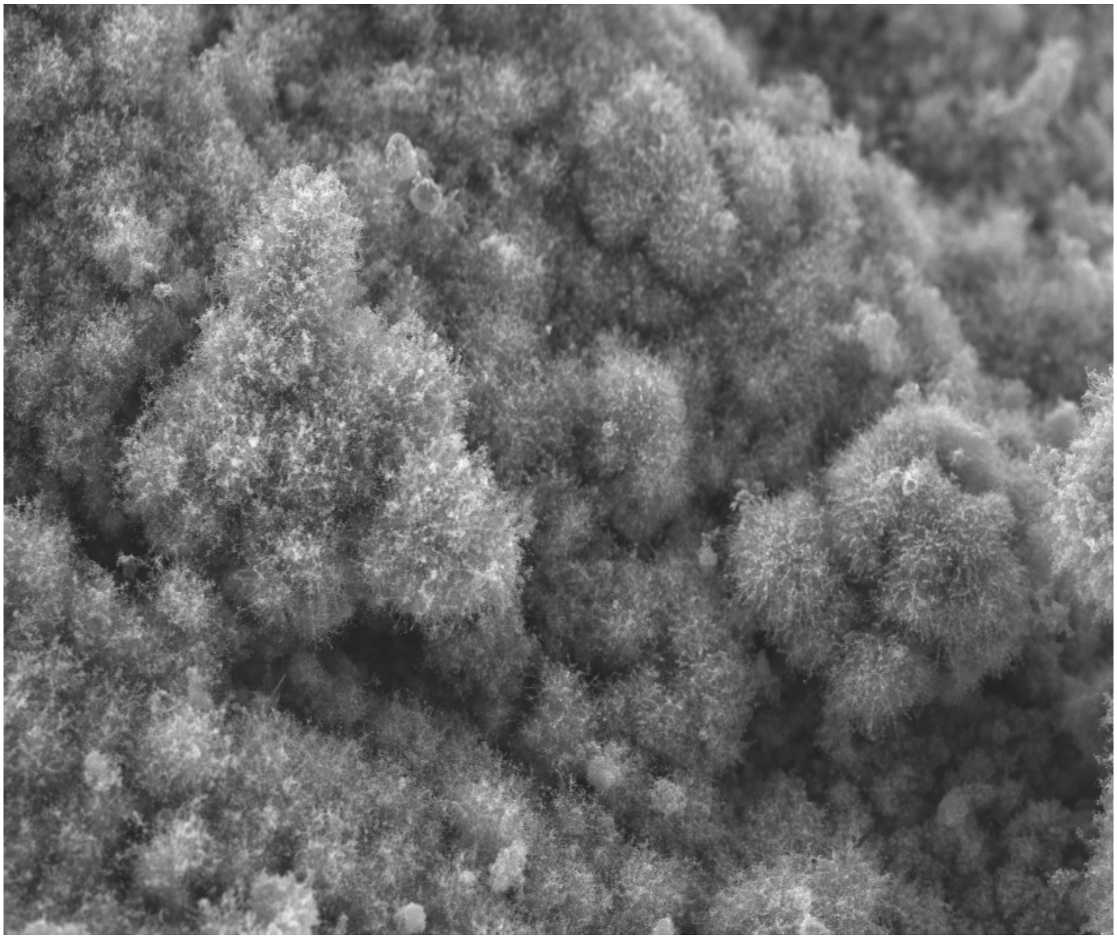

**Figure S1.** SEM micrographs of carbon nanotube (CNT) clusters grown using waste polyethylene as carbon source and commercial palladium on carbon (30% Pd) showing a "St Augustine grass" type of morphology.

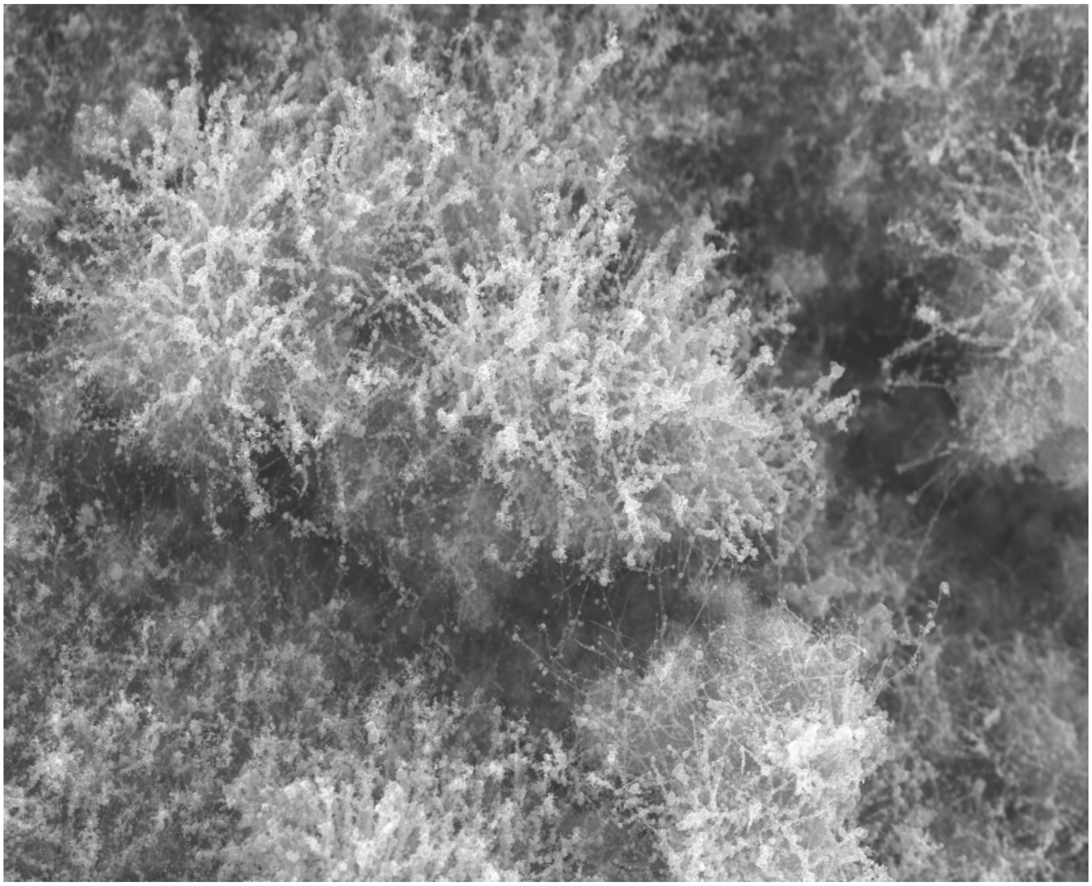

**Figure S2.** SEM micrographs of CNT clusters grown using waste polyethylene as carbon source and commercial palladium on carbon (30% Pd) showing a "St Augustine grass" type of morphology.

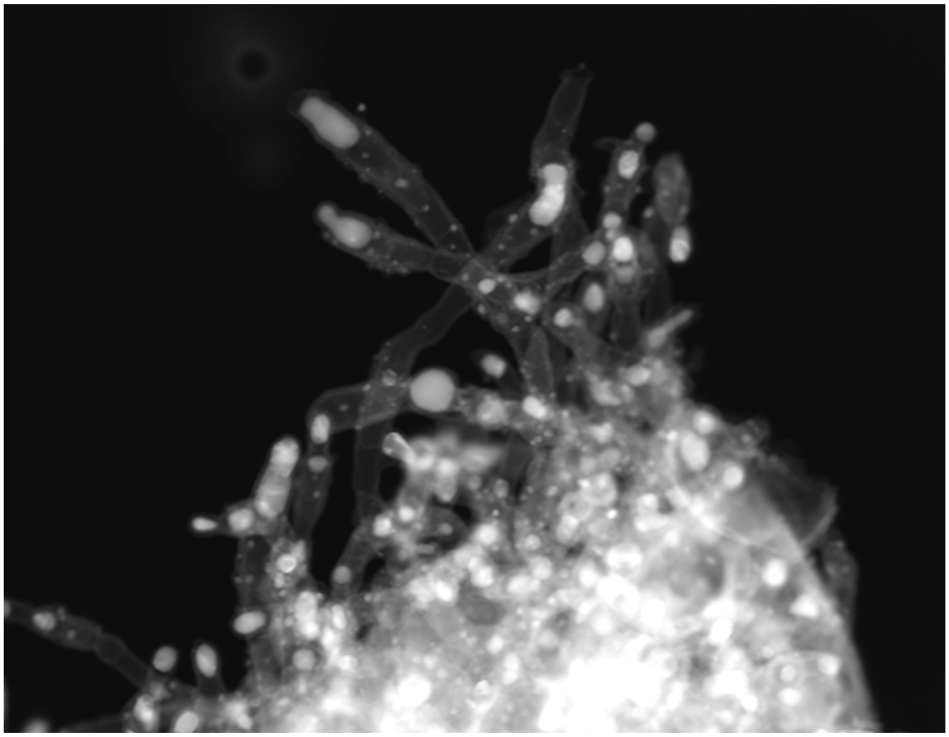

**Figure S3.** HAADF dark field image corresponding of G-NCNT-Fe corresponding to Figure 1c.
